# Military-Type Workload and Footwear Alter Lower Extremity Muscle Activity during Unilateral Static Balance: Implications for Tactical Athletic Footwear Design

**DOI:** 10.3390/sports8050058

**Published:** 2020-04-28

**Authors:** Christopher M. Hill, Hunter DeBusk, Adam C. Knight, Harish Chander

**Affiliations:** 1 Department of Kinesiology and Physical Education, Northern Illinois University, Dekalb, IL 60115, USA; 2 Applied Cognitive Sciences, Mica, WA 99023, USA; hunter@acsciences.com; 3 Department of Kinesiology, Mississippi State University, Starkville, MS 39762, USA; AKnight@colled.msstate.edu

**Keywords:** footwear, balance, military, electromyography

## Abstract

Maintaining upright standing balance is critical for military personal. The impact of military footwear and occupation-related fatigue on muscle activity during balance performance has been previously documented. However, the current literature has not provided a muscle activation profile of the lower extremity during challenging conditions such as unilateral balance trials. Twenty-two recreationally active male participants (age: 22.2 ± 2.7 years; height: 177 ± 6.8 cm; mass: 79.8 ± 9.7 kg) donned two styles of military footwear (minimalist and standard) and performed a military style workload. Unilateral static balance was accessed before (PRE) and after (POST) the workload as surface electromyography was recorded on the right lower extremity. This study found that the minimalist footwear increased muscle activation prior to the workload compared to the standard footwear (co-contraction index mean difference: 0.149), whereas the standard footwear increased muscle activity after the workload (co-contraction index mean difference: 0.097). These findings suggest that footwear design characteristics affect lower extremity muscle activity differently depending on the workload condition. These findings intend to aid in the design of military footwear to maximize balance performance in a military population.

## 1. Introduction

Tactile athlete is a term used to describe persons working in service professions that require high levels of physical fitness and performance to meet the demands of their occupational task [1]. Military personnel fit into this category given their consistent exposure to hazardous occupational settings and intense workloads. Military personal are exposed to environments with uneven and narrow ground surfaces and conditions with low light while carrying substantial loads such as military rucksacks. These precarious work environments increase the risk for the development of musculoskeletal injuries in this population. Among the recently reported injury types for military service personal, falls account for 21.3% of non-battle injuries [2]. The prevalence of these incidents suggests military personnel are predisposed to suffering losses of balance stemming from the demands of their occupation.

One reason military personnel are unable to arrest a loss of balance may be due to inadequate postural control responses stemming from a combination of intrinsic human factors and extrinsic environmental factors. The most influential human factor is skeletal muscle fatigue of the lower extremity. The mechanistic goal of upright human standing is to maintain the body’s center of mass within the base of support. However, maintaining postural stability and equilibrium is challenging due to the arrangement of the center of mass in relation to the base of support. This arrangement requires constant maintenance from the central and peripheral nervous systems, which detects and integrates sensory stimuli and reacts with reflexive responses from the lower extremity muscles to compensate for the disruption to postural equilibrium [3]. This maintenance of upright standing requires relatively little muscle activation and co-contraction between lower extremity muscle agonist–antagonist pairs in acute conditions [3]. Multiple studies have documented that changes to lower extremity muscle activity is associated with poorer balance maintenance after performing fatiguing workloads [3,4,5,6]. Bodily fatigue changes the activation pattern of lower extremity muscles by increasing co-contraction and muscle excretion, to minimize the sway of the center of mass to maintain upright stability [5,6]. This change to the lower extremity muscle activation pattern is linked to changes in motor control properties of the muscle and changes to sensory feedback for the periphery [3,4]. Specific to the military occupational settings, balance maintenance and lower extremity muscle activity during bilateral static balance has been shown to change after a fatiguing military style workloads [6,7,8].

In occupational settings, footwear is a crucial external factor that alters lower extremity muscle activity during upright standing. Usage and access to occupationally appropriate footwear is critical to balance maintenance, as footwear is the primary mediator between the human body and the physical environment [5]. Occupational footwear characteristics can act to decrease metabolic expenditure, prevent slips, provide support to the ankle, and guard the base of the foot from penetrating objects. Numerous studies have noted that footwear characteristics such as heel to toe drop, boot shaft height and stiffness, and midsole cushioning influence the muscle activation pattern found in the lower extremity during the assessment of balance [9,10,11,12,13]. For instance, Böhm and Hösl (2008) noted that stiffer boot shafts created increased co-contraction between plantarflexors and dorsiflexors during over ground walking [9]. Moreover, thick cushioned midsoles interfere thick midsoles decrease somatosensory feedback from cutaneous receptors of the foot at the base of the foot resulting in changes in activation magnitude of postural muscles of the lower extremity [13]. 

The parameters of military footwear are regulated under the United States Army regulation 670-1 (AR670-1). However, there is a diverse selection of military boots that meet the requirements. Previous studies have demonstrated that various characteristics among military footwear alter upright postural control [14,15]. Primarily, the investigations of balance maintenance in military footwear have been conducted in bilateral static stance [6,7]. Only one of the associated studies explored the effect different military footwear types have on unilateral static balance performance. DeBusk et al., (2018) reported that a minimalist style military boot outperformed the standard style military boot in unilateral balance assessments, both before and after a simulated physiological workload [7]. However, the study did not examine how military footwear and workload impacted the lower extremity muscle activation profile during unilateral static balance. Though there is a considerable amount of literature discussing balance performance comparing standard military footwear to minimalist style footwear, there remains a dearth in the literature concerning the lower muscular activation profile during a unilateral static balance task while wearing either type of military footwear in a trained population. An examination of this facet would provide a greater understanding of how these types of military footwear influence postural control under challenging conditions, which would more closely resemble real world conditions of military personal (e.g., narrow terrain with uneven surfaces). This would further aid in the design of military footwear to meet the needs of a challenging occupational environment and enhance the safety of military tactical athletes. Thus the purpose of the study is to assess the level of lower extremity muscular activity using surface electromyography (EMG) during unilateral balance tasks before and after a military style physiological workload, in two different AR670-1 compliant boots: Belleville 310ST hot weather standard tactical boot (STD) and Belleville TR101 MiniMil ultra-light minimalist style military boot (MIN). We hypothesized that design characteristics of the standard footwear would aid in unilateral stability prior to performing an occupational workload, but exacerbate the effects of bodily fatigue resulting in increased muscle exertion. 

## 2. Materials and Methods

The study was conducted in a repeated measures, pre-test-post-test design, with twenty-two healthy male adults (age: 22.2 ± 2.7 years; height: 177 ± 6.8 cm; mass: 79.8 ± 9.7 kg) with no history of musculoskeletal, orthopedic, neurological, cardiovascular, and vestibular abnormalities serving as participants. Participant’s physical fitness status was also above recreationally trained (>3–4 days/week with consistent aerobic and anaerobic training for at least the last 3 months) and were naïve to military footwear and load carriage. A naïve sample was selected to mimic incoming military service members to basic training where a substantial number of musculoskeletal injuries occur [16]. All participants provide an informed consent and all data collection procedures were approved by the University’s Institutional Review Board. Data collection procedures included an initial familiarization of the balance and workload protocols. The experimental testing was conducted on two separate days separated by at least 72 h with a counter balanced footwear assignment (i.e., participants wore either the standard or minimalist during the first testing session and other footwear during the second testing session) to remove order effects. A description of footwear characteristics can be found in Table 1. 

Participants performed three trials of 20-second unilateral static balance trials while standing on top of a force plate, with their right leg without an external load, before (PRE) and after (POST) a military type workload [4]. Unilateral static balance was accessed in three sensory conditions: eyes-open (EO), eyes-closed (EC), and eyes-open foam (EOF). The foam surface condition utilized a 6.35 cm thick Airex Balance Pad (Sins, Switzerland) to create a challenging sensory condition to mimic unstable terrain. An eyes-closed foam condition was collected, however, due to the low number of successful trials this condition was removed from the analysis.

The workload utilized a modified Bruce protocol, adapted from DeMaio et al., (2009), that was performed with a 16 kg backpack, adapted from Strube et al., (2017) [17]. This protocol was conducted on a treadmill and consisted of 3 min increment periods starting at 4.83 km/h at 0% grade, and increasing to 5.632 km/h and 6.44 km/h at 0% grade until minute 9, following which the grade was increased by 5% every 3 min up to 18 min [8]. This particular protocol was chosen to simulate a high intensity load carrying task experienced by military personnel. During the workload, subjective ratings of perceived exertion (RPE) were collected every three min from all participants, using the Borg’s 6-20 RPE scale [18]. All participants were verbal encouraged to perform at maximal effort. The workload was completed when the participant reached twenty-one min or until the participant’s volitional exhaustion, where time spent on the treadmill was recorded. After the completion of the workload, a three minute walking cool-down was performed before the completion of the post-workload unilateral static balance trials. 

Muscle activity was collected on the right lower extremity’s medial gastrocnemius (Plantar Flexor-PF), tibialis anterior (Dorsi Flexor-DF), peroneus longus (Evertor-EV), and tibialis posterior (Invertor-IN) using MP150 EMG system (BIOPAC, Aero Camino Goloeta, CA, USA). The EMG data collected was sampled at 1000 Hz. The raw EMG data was bandpass filtered at 20–250 Hz and full-wave rectified before exporting for analysis. Mean EMG data for all four muscles (mean PF, mean DF, mean IN, mean EV) were calculated for all unilateral static standing trials, followed by calculations of root-mean squared (RMS) EMG (RMS PF, RMS DF, RMS IN, RMS EV). Co-contraction between the muscle pairs were calculated using the Co-Contraction Index (CCI) for the agonist-antagonist pairs of DF & PF and IN & EV using mean muscle activity using Equation (1) [19]:

(1) $$\left(EMG_{Least}+EMG_{Most}\right)\times EMG_{Least}/EMG_{Most}$$

A 2 (Footwear: STD × MIN) × 2 (Workload: PRE × POST) repeated measures ANOVA was used to analyze all of the above described EMG dependent variables. A paired samples t-test was conducted to test for footwear differences in time on treadmill. A Wilcoxon signed-rank test was used to test for differences between footwear conditions on the final rating of perceived exertion value provided at the conclusion of the workload. All analysis was conducted with at alpha level of 0.05 using SPSS version 21. Post-hoc pairwise comparisons were performed using a Bonferroni correction if interaction/main effects were found.

## 3. Results

### 3.1. Mean Muscle Activity

Mean muscle activity changes were found in relationship to both footwear and workload in the plantarflexors and invertors across sensory conditions. Footwear differences were found for EOF PF [F (1, 21) = 7.127, *p* = 0.014, $\eta^{2}$_p_ = 0.234], EC IN [F(1, 21) = 4.412, *p* = 0.048, $\eta^{2}$_p_ = 0.185], and EOF IN [F(1, 21) = 4.853 *p* = 0.039, $\eta^{2}$_p_ =0.188]. The minimalist footwear demonstrated greater mean muscle activity compared to the standard footwear for EOF PF (mean difference: 0.21, 95% confidence intervals: 0.004–0.035), EC IN (mean difference: 0.009, 95% confidence intervals: 0.001–0.017), and EOF IN (mean difference: 0.008, 95% confidence intervals: 0.001–0.015). 

**Figure 1:**
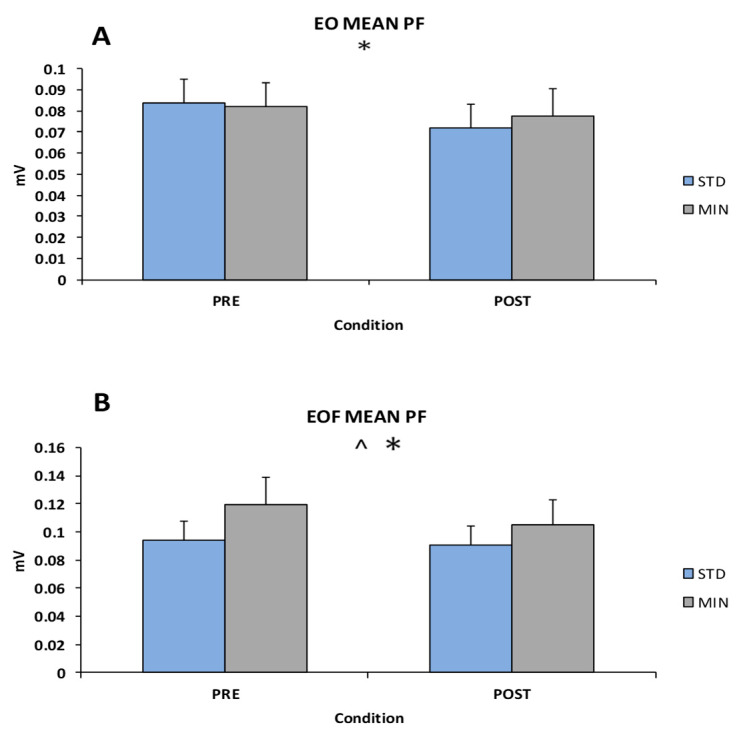
(**A**) Eyes-open (EO) mean muscle activity of the plantarflexor (PF) * denotes significant main effect for footwear at *p* < 0.05. (**B**) Eyes-open Foam (EOF) mean muscle activity of the plantarflexor (PF) * denotes a significant main effect for footwear at *p* < 0.05 and ^ denotes a significant main effect for workload at *p* < 0.05.

Workload differences were found for EO PF [F(1, 21) = 4.542, *p* = 0.045, $\eta^{2}$_p_ = 0.376), EOF PF [F(1, 21) = 4.958, *p* = 0.037, $\eta^{2}$_p_ = 0.191], EC IN [F(1, 21) = 6.423, *p* = 0.019, $\eta^{2}$_p_ = 0.234], and EOF IN [F(1, 21) = 6.282, *p* = 0.02, $\eta^{2}$_p_ = 0.23]. PRE demonstrated greater mean muscle activity compared to POST for EO PF (mean difference: 0.008, 95% confidence intervals: 0.001–0.016) (Figure 1A), EOF PF (mean difference: 0.009, 95% confidence intervals: 0.001–0.017) (Figure 1B), EC IN (mean difference: 0.008, 95% confidence intervals: 0.002–0.015), and EOF IN (mean difference: 0.008, 95% confidence intervals: 0.001–0.015). Descriptive statistics of these variables are listed in Table 2. 

### 3.2. Root-Mean Square Muscle Activity

Similar changes were noted for root-mean square muscle activity. Footwear main effects were also found for EC IN [F(1, 21) = 6.628, *p* = 0.018, $\eta^{2}$_p_ = 0.240], EOF PF [F(1, 21) = 6.953, *p* = 0.015, $\eta^{2}$_p_ = 0.249], and EOF IN [F(1, 21) = 13.078, *p* = 0.002, $\eta^{2}$_p_ = 0.384]. The minimalist footwear demonstrated greater root-mean square muscle activity compared to the standard footwear for EC IN (mean difference: 0.004, 95% confidence intervals: 0.001–0.007), EOF PF (mean difference: 0.031, 95% confidence intervals: 0.006–0.055), and EOF IN (mean difference: 0.021 95% confidence intervals: 0.009–0.032) conditions. Descriptive statistics of these variables are listed in Table 3. 

Workload differences were found for EC PF [F(1, 21) = 4.833, *p* = 0.039, $\eta^{2}$_p_ = 0.187], and EOF IN [F(1, 21) = 9.685, *p* = 0.005, $\eta^{2}$_p_ = 0.316]. PRE demonstrating greater root-mean square muscle activity compared to POST for EC PF (mean difference: 0.005, 95% confidence intervals: 0.001–0.009) and EOF IN (mean difference: 0.016, 95% confidence intervals: 0.005–0.026).

### 3.3. Co-Contraction Index

**Figure 2:**
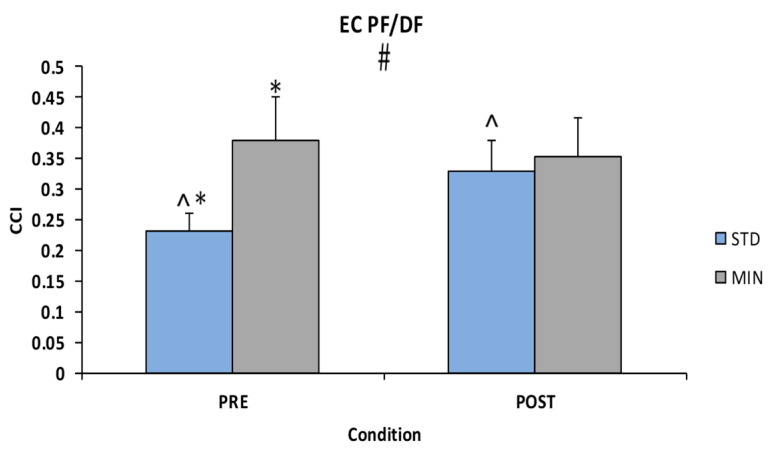
Eyes-close (EC) co-contraction index (CCI) of the plantarflexor (PF) dorsiflexor (DF) muscle pair. # denotes a significant interaction with * denoting a significant simple effect for footwear and ^ denoting a significant simple effect for time.

Co-contraction between plantarflexors and dorsiflexors was altered by both footwear and workload. A footwear and workload interaction was found for EC PF/DF CCI [F(1, 21) = 5.899, *p* = 0.024, $\eta^{2}$_p_ = 0.219] (Figure 2). Tests of simple main effects revealed significant differences with higher CCI in minimalist footwear during PRE compared to standard footwear (mean difference: 0.149, 95% confidence intervals: 0.027–0.271) and for standard footwear with higher CCI in POST compared to PRE (mean difference: 0.097, 95% confidence intervals: 0.111–0.185) significant at *p* = 0.019 and *p* = 0.031 respectively. No significant differences for footwear or workload were found in IN-EV muscle pairs across sensory conditions. Descriptive statistics of these variables are listed in Table 4. 

### 3.4. Workload Descriptives

**Figure 3:**
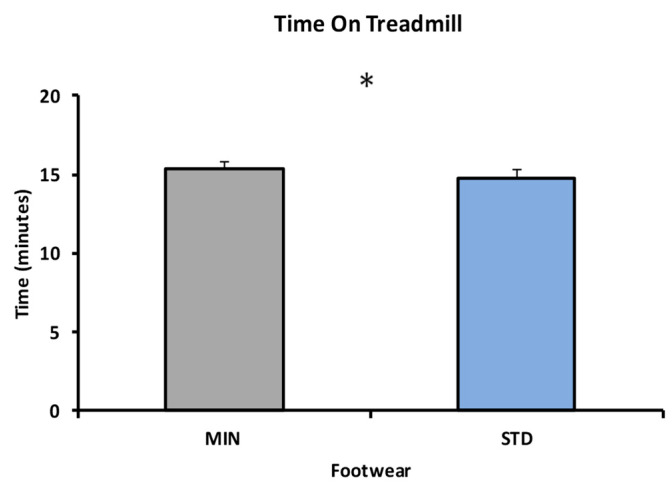
Time spent on the treadmill in min between * denoting a significant main effect for footwear at *p* < 0.05.

Significant footwear difference was found in time on treadmill (t(21) = 2.733, *p* = 0.012, Cohen’s d = 0.583) with MIN (15.39 ± 2.13 min) having a longer workload duration than STD (14.79 ± 2.38 min) (Figure 3). 

No signficant differcenes were found between standard (18.95 ± 2.193 RPE) and minimlist (18.96 ± 0.99 RPE) footwear on final RPE (W = 9.00, *p* = 0.063). 

## 4. Discussion

Unilateral static balance provides a challenging environment to maintain upright standing. A narrowed base of support and a lower amount of muscular torque to counteract shifts in the center of mass limits the effectiveness of the postural control loop [20]. Additionally, manipulating external and internal factors, such as footwear and muscular fatigue, increases the stress placed on the postural control loop during unilateral standing. Often, military service members are exposed to environments that tax the postural control system, such as uneven and narrow terrain. The footwear worn by military personal are the primary mediators between their bodies and the occupational environment. Thus, it is of grave importance that footwear worn by these tactical athletes is optimally designed for these hazardous scenarios. The goal of this study was to determine the impact of military style footwear and workload on lower extremity muscle activity during unilateral static balance testing. We found that minimalist style military footwear demonstrated higher muscle activity compared to the standard military footwear in acute conditions. Additionally, the findings of this study point toward footwear characteristics of the standard military footwear increasing co-contraction as a result of the simulated occupational workload during unilateral static standing. 

Before the simulated occupational workload, mean and root-mean square muscle activity surrounding the lower extremity was higher than after the workload. Gribble and Hertel (2004) noted localized fatigue to the musculature surrounding the ankle impaired unilateral static balance [21]. It was further suggested by Yaggie and McGregor (2002) that fatigue of the ankle musculature compromised the proprioceptive system, which resulted in a greater displacement of the center of pressure in the sagittal plane [22]. The workload utilized during our study was conducted on a treadmill that primarily featured the sagittal plane movements of plantarflexion and dorsiflexion at the ankle joint. Our results suggest that the occupational style workload altered the proprioception of the plantar flexors, resulting in lower muscle exertion after the workload. Such changes may limit the neuromuscular system from providing an adequate response to a postural disruption, which may increase the risk of a fall event. 

We found greater co-contraction in the eyes-closed condition supporting previous literature that reported greater co-contraction in altered visual information. Vuillerme and colleagues (2006) found that during static standing after an ankle fatigue protocol, limiting visual information was detrimental to balance performance [23]. In the case of our study, once visual feedback was altered, increased co-contraction of PF-DF pair was needed to maintain balance. Greater co-contraction of this agonist–antagonist muscle pair was exhibited during pre-workload of the eyes closed condition when participants wore the minimalist footwear compared to the standard footwear. However, during post workload, a similar co-contraction index was found when participants wore either boot. Such results suggest that while wearing the standard footwear, participants may have increased their reliance on proprioceptive information from the muscles around the ankle joint in order to compensate for the lack of visual information and muscular fatigue. 

The minimalist footwear increased root-mean square muscle activity while standing on the foam surface. The minimalist footwear features an outsole surface area that is 53.6 cm^2^ smaller than that of the standard footwear. A larger footwear outsole increases the overall size of the base of support, which decreases postural instability [7,24]. When the participants were exposed to the unstable foam surface, the large outsole surface of the standard footwear increased the limits of stability thus allow for more stable upright standings. The narrow outsole of the minimalist footwear does not provide a suitable base of support when in contact with an unstable surface resulting in greater muscle exertion to maintain the body’s center of mass within the base of support. Thus, a large outsole could be considered a positive design characteristic that is best suited to counteract the unstable surfaces.

The minimalist footwear demonstrated a higher co-contraction index in the PF-DF pair before the military workload compared to the standard footwear. The minimalist boot features a thin pliable shaft in comparison to the standard footwear, which features a stiff thick boot shaft suggesting that in acute conditions the thick boot shaft decreases muscular exertion of the muscles around the ankle by decreasing the range of motion in the anterior and posterior directions. As a result, the thin, less stiff boot-shafts of the minimalist footwear allows for greater range of motion at the ankle in dorsiflexion and plantarflexion [9]. However, after the workload, in order to compensate for fatigue, co-contraction index increased to stabilize the ankle joint while donning the standard footwear. Thus, after the workload, the boot shafts similarly affected the surrounding ankle musculature. The mass of the standard footwear also influences this finding. Previous literature has displayed an increase in the rate of energy expenditure and muscular fatigue when footwear mass is increased [25]. The standard footwear is 300 g heavier than minimalist footwear, suggesting that the increase in co-contraction index after the occupational workload may be a result of the increased standard footwear mass, while the lighter minimalist footwear maintained a similar co-contraction index to its pre-workload condition. Thus, the mass of the standard footwear increased the rate of the fatigue and eliminated the positive affects noted during the pre-workload balance testing. Under fatiguing conditions, which military personal are constantly exposed to, the lower mass of the minimalist footwear would limit the effects of fatigue on balance performance. 

The final RPE value did not differ between footwear conditions, suggesting the footwear itself did not affect perceived exertion during the load carriage exercise. However, taking this finding with the time spent of the treadmill suggests that, while wearing the minimalist footwear, participants have a higher performance with similar exertion levels. Overall, donning the standard footwear was more fatiguing during a shorter period of time, though the perceived exertion was similar.

## 5. Conclusions

The design of tactical military footwear influences the balance performance before and after an occupational workload. Our findings demonstrate that footwear characteristics of the standard footwear have positive design facets that are beneficially to unilateral static balance in acute conditions. However, the minimalist style footwear limited the impact of a fatiguing workload on unilateral static balance performance, which would be more appropriate for a physically taxing occupational environment of the military tactical athlete.

## Figures and Tables

**Table 1:** Footwear Design Characteristics.

Footwear Design Characteristics	Standard Tactical Boot (STD)	Minimalist Tactical Boot (MIN)
Mass	801.13 ± 40.4 g	500.13 ± 24.1 g
Sole surface area	288.6 ± 24.1 cm^2^	235 ± 8.21 cm^2^
Boot shaft height	20 cm	20 cm
Heel-toe drop	18 mm	2 mm
Heel thickness	3.31 ± 0.17 cm	0.97 ± 0.06 cm
Nature of midsole	Soft and cushioned	Hard and thin
Midsole hardness (Shore A)	66.0 HA	83.2 HA

**Table 2:** Mean Muscle Activity.

	Pre-Workload		Post-Workload	
	STD	MIN	STD	MIN
Condition	MEAN ± SD	MEAN ± SD	MEAN ± SD	MEAN ± SD
**EO PF**	0.0835 ± 0.0529 ^	0.0820 ± 0.0532 ^	0.0719 ± 0.0519	0.0777 ± 0.0612
**EO EV**	0.0810 ± 0.0337	0.0972 ± 0.0721	0.0732 ± 0.0296	0.0866 ± 0.0504
**EO DF**	0.0502 ± 0.0227	0.0541 ± 0.0313	0.0543 ± 0.0305	0.0490 ± 0.0304
**EO IN**	0.0502 ± 0.0227	0.0541 ± 0.0313	0.0543 ± 0.0305	0.0490 ± 0.0304
**EC PF**	0.1654 ± 0.1071	0.1532 ± 0.1197	0.1384 ± 0.0927	0.1510 ± 0.1213
**EC EV**	0.1368 ± 0.0509	0.1423 ± 0.0841	0.1274 ± 0.0524	0.1479 ± 0.0867
**EC DF** §	0.1654 ± 0.1071 +	0.1532 ± 0.1197	0.1384 ± 0.0927 +	0.1510 ± 0.1213
**EC IN**	0.0901 ± 0.0472	0.0889 ± 0.0419 *	0.0950 ± 0.0610 ^	0.0883 ± 0.0572 ^ *
**EOF PF**	0.0939 ± 0.0617 ^	0.1195 ± 0.0912 * ^	0.0906 ± 0.0643	0.1047 ± 0.0824 *
**EOF EV**	0.0861 ± 0.0292	0.1043 ± 0.0585	0.0868 ± 0.0363	0.0932 ± 0.0470
**EOF DF**	0.0638 ± 0.0507	0.0869 ± 0.0864	0.0527 ± 0.0450	0.0470 ± 0.0546
**EOF IN**	0.0616 ± 0.0261 ^	0.0531 ± 0.0321 * ^	0.0702 ± 0.0416	0.0620 ± 0.0402 *

Descriptive statistics (Mean ± Standard Deviation) for Standard Boot (STD) and Minimalist Boot (MIN) from static balance muscle activity during Eyes-Open (EO), Eyes Closed (EC), and Eyes-Open Foam (EOF) testing conditions. § denotes significant interaction with + denoting significant simple effect for footwear at *p* < 0.05. * denotes significant main effect for footwear, ^ denotes significant main effect for workload at *p* < 0.05.

**Table 3:** Root-Mean Squared Muscle Activity.

	Pre-Workload		Post-Workload	
	STD	MIN	STD	MIN
Condition	MEAN ± SD	MEAN ± SD	MEAN ± SD	MEAN ± SD
**EO PF**	0.1298 ± 0.0771	0.1302 ± 0.0839	0.1144 ± 0.0767	0.1205 ± 0.0923
**EO EV**	0.1502 ± 0.0676	0.1827 ± 0.1353	0.1373 ± 0.0585	0.1670 ± 0.1025
**EO DF**	0.0854 ± 0.0442	0.1135 ± 0.0939	0.0868 ± 0.0637	0.0952 ± 0.0601
**EO IN**	0.0796 ± 0.0380	0.0864 ± 0.0527	0.0857 ± 0.0530	0.0745 ± 0.0483
**EC PF**	0.2306 ± 0.1149 ^	0.2112 ± 0.1361 ^	0.2020 ± 0.1124	0.2071 ± 0.1557
**EC EV**	0.2452 ± 0.0987	0.2557 ± 0.1636	0.2267 ± 0.1013	0.2666 ± 0.1672
**EC DF**	0.0071 ± 0.0079	0.0061 ± 0.0044	0.0058 ± 0.0052	0.0611 ± 0.0052
**EC IN**	0.1494 ± 0.0845	0.1439 ± 0.0715 *	0.1502 ± 0.1054	0.1440 ± 0.0967 *
**EOF PF**	0.1483 ± 0.0925	0.1873 ± 0.1420 *	0.1466 ± 0.1001	0.1688 ± 0.1280 *
**EOF EV**	0.1559 ± 0.0567	0.1951 ± 0.1191	0.1571 ± 0.0659	0.1794 ± 0.1020
**EOF DF**	0.1179 ± 0.0892	0.1621 ± 0.1543	0.1055 ± 0.0908	0.1444 ± 0.0984
**EOF IN**	0.0970 ± 0.0450 ^	0.1198 ± 0.0739 * ^	0.0834 ± 0.0551	0.1017 ± 0.0684 *

Descriptive statistics (Mean ± Standard Deviation) for Standard Boot (STD) and Minimalist Boot (MIN) from static balance muscle activity during Eyes-Open (EO), Eyes Closed (EC), and Eyes-Open Foam (EOF) testing conditions. * denotes significant main effect for footwear, ^ denotes significant main effect for time at *p* < 0.05.

**Table 4:** Co-contraction Index.

	Pre-Workload		Post-Workload	
	STD	MIN	STD	MIN
Condition	MEAN ± SD	MEAN ± SD	MEAN ± SD	MEAN ± SD
**PF/DF EO**	0.0670 ± 0.0400	0.0844 ± 0.0766	0.0621 ± 0.0480 ^	0.0712 ± 0.0427 ^
**PF/DF EC** §	0.2300 ± 0.1393 # +	0.3785 ± 0.3290 #	0.3274 ± 0.2444 +	0.3522 ± 0.2939
**PF/DF EOF**	0.0896 ± 0.0632	0.1670 ± 0.1870	0.1054 ± 0.1263	0.1162 ± 0.0829
**IN/EV EO**	0.0807 ± 0.0380	0.2156 ± 0.4019	0.1040 ± 0.0718	0.1339 ± 0.1451
**IN/EV EC**	0.2301 ± 0.2616	0.1803 ± 0.1188	0.1864 ± 0.1282	0.1626 ± 0.1076
**IN/EV EOF**	0.1037 ± 0.0441	0.1137 ± 0.0825	0.1068 ± 0.0808	0.1090 ± 0.0729

Descriptive statistics (Mean ± Standard Deviation) for Standard Boot (STD) and Minimalist Boot (MIN) from static balance muscle activity during Eyes-Open (EO), Eyes Closed (EC), and Eyes-Open Foam (EOF) conditions. § denotes significant interaction with # denoting significant simple effect for time, + denoting significant simple effect for footwear at *p* < 0.05. * denotes significant main effect for footwear, ^ denotes significant main effect for time at *p* < 0.05.
